# Acute effects of moderate-intensity continuous physical exercise performed with different amounts of muscle mass on executive function in healthy young adults: a randomized trial

**DOI:** 10.17179/excli2023-6434

**Published:** 2023-09-25

**Authors:** Marcos José Morais, Vinnycius Nunes de Oliveira, Ricardo Borges Viana, Marilia Santos Andrade, Rodrigo Luiz Vancini, Ricardo Mario Arida, Gustavo De Conti Teixeira Costa, Mario Hebling Campos, Carlos Alexandre Vieira, Katja Weiss, Beat Knechtle, Claudio Andre Barbosa de Lira

**Affiliations:** 1 Faculdade de Educação Física e Dança, Universidade Federal de Goiás, Goiânia, Brazil; 2 Instituto de Educação Física e Esportes, Universidade Federal do Ceará, Fortaleza, Brazil; 3 Departamento de Fisiologia, Universidade Federal de São Paulo, São Paulo, Brazil; 4 Centro de Educação Física e Desportos, Universidade Federal do Espírito Santo, Vitória, Brazil; 5 Institute of Primary Care, University of Zurich, Zurich, Switzerland; 6 Medbase St. Gallen, Switzerland

**Keywords:** physical activity, cognitive function, cognition, attention, inhibitory control

## Abstract

We examined the effect of amount of muscle mass involved in moderate-intensity continuous physical exercise on executive function. To this end, fifty-five participants completed two acute physical exercise sessions on an airbike ergometer using the upper and lower limbs simultaneously and only the upper limbs, and a resting control session in a randomized order. The physical exercise session lasted 30 min and was performed at moderate intensity (between 64 %-76 % of maximal heart rate evaluated in graded maximal exercise testing). Participants took the Stroop test (congruent and incongruent trials) before and after the sessions to assess executive performance. For the congruent trial, both physical exercise interventions improved executive function performance (pre vs. post, p-value = 0.002 and 0.003 for physical exercise with upper limbs and physical exercise with upper and lower limbs, respectively). Furthermore, executive function performance was higher after the physical exercise interventions than after the control session (p-value = 0.002 and 0.004 for physical exercise with upper limbs and physical exercise with upper and lower limbs, respectively). For the incongruent trial, both physical exercise interventions also improved executive function performance (pre vs. post, p-value < 0.001 for physical exercise with upper limbs and physical exercise with upper and lower limbs, respectively). However, there were no significant differences after both physical exercise interventions and resting control session (p-value = 0.175). Executive function (congruent trial) was positively impacted by acute aerobic physical exercise regardless of the amount of muscle mass involved (upper limbs or upper plus lower limbs). Therefore, we recommend aerobic physical exercise with less or more muscle mass involved to improve cognitive function.

## Introduction

Executive functions are a set of cognitive processes that, in an integrated manner, enable the individual to direct behaviors to achieve goals, evaluate the efficiency and adequacy of these behaviors, abandon ineffective strategies in favor of more efficient ones, and solve immediate, medium and, long-term problems (Malloy-diniz et al., 2010[28]). 

The core elements of executive functions are inhibitory control, working memory, and cognitive flexibility (Diamond, 2013[17]). Inhibitory control is a mechanism for inhibiting irrelevant or distracting stimuli and/or inappropriate responses to maintain attention in a particular focus (Diamond, 2013[17]). Working memory can be defined as the ability to temporarily store information and mentally work with it (Diamond, 2013[17]). Furthermore, cognitive flexibility can be defined as the ability to dynamically alter the course of cognitive processing in line with environmental demands (Diamond, 2013[17]; Borkertienė et al., 2015[8]; Lin et al., 2018[26]; Xue et al., 2018[49]). Executive functions are linked to several important aspects of daily life, such as academic and professional success, psychological health, and social development (Diamond, 2013[17]; Borkertienė et al., 2015[8]; Gu et al., 2019[22]; Stenling et al., 2019[40]). Moreover, impaired cognitive functions have been associated with aging (Park et al., 2001[35]; Hedden and Gabrieli, 2004[23]; Smiley-Oyen et al., 2008[39]), which negatively affects quality of life (Smiley-Oyen et al., 2008[39]). Therefore, strategies to minimize cognitive decline or even improve cognitive performance are necessary.

Aerobic physical exercise can positively affect executive function performance at practically all ages (Best, 2010[6]; Verburgh et al., 2014[48]; Ludyga et al., 2016[27]; Xue et al., 2018[49]). Additionally, physical exercise can minimize the cognitive decline associated with aging (Ludyga et al., 2016[27]). An advantage of physical exercise is that it is an intervention that rarely causes side effects and can prevent and treat various clinical situations simultaneously.

Several mechanisms have been proposed to explain the positive effects of aerobic physical exercise on executive functions. Briefly, these mechanisms can be divided into biological and psychological mechanisms. Biological mechanisms include increased cerebral blood flow (Querido and Sheel, 2007[36]; Best, 2010[6]; Verburgh et al., 2014[48]; Vazou et al., 2016[47]), angiogenesis (Swain et al., 2003[41]; Ding et al., 2006[18]; Best, 2010[6]; Verburgh et al., 2014[48]; Vazou et al., 2016[47]), increased availability of oxygen to the brain (Curlik and Shors, 2013[14]), and changes in plasma levels of catecholamines, adrenocorticotropic hormone, vasopressin, and β-endorphin, which may reflect increased activation of the central nervous system (Verburgh et al., 2014[48]). Additionally, several neurotrophic factors, such as brain-derived neurotrophic factor (BDNF), nerve growth factor, vascular endothelial growth factor, granulocyte colony-stimulating factor, and the endocannabinoid system, are regulated by physical exercise and play an important role in neurogenesis, neuron survival and cognitive functioning (Curlik and Shors, 2013[14]; Fagundo et al., 2013[19]; Verburgh et al., 2014[48]), and synaptogenesis (Mekari et al., 2019[31]). Psychological mechanisms refer to the fact that physical exercise induces excitement (at adequate levels) that can optimize the allocation of mental resources, thus facilitating the cognitive process (Mandolesi et al., 2018[29]).

Despite advances in understanding the effects of acute and chronic physical exercise, it is still unclear how the amount of muscle mass involved in physical exercise affects these functions. Physical exercise performed with different amounts of muscle mass elicits different physiological responses (Nagle et al., 1984[34]). Physical exercises performed with the upper limbs seem to promote greater cerebral blood flow (Dalsgaard et al., 2004[15]) and greater production of catecholamines than physical exercise performed with the lower limbs (Davies et al., 1974[16]).

However, to the best of our knowledge, only one study has investigated the effect of the amount of muscle mass involved in performing physical aerobic exercise on cognition. Hill et al., (2019[24]) investigated the maximal and submaximal effects of cycling performed with the upper limbs and cycling performed with the lower limbs on executive functions in healthy men. They found that both physical exercise intervention at an intensity of 50 % of the maximum workload and physical exercise intervention at maximal intensity performed with upper limbs improved cognitive performance. However, Hill et al., (2019[24]) did not include a group without physical exercise and evaluated only men in their study. Therefore, a study that investigates women and includes a resting control session can reduce bias from sex and random error. Thus, the primary objective of this study was to investigate the effect of the amount of muscle mass involved in acute moderate-intensity continuous aerobic physical exercise on executive function. Second, we compared the rate of perceived exertion (RPE), maximal heart rate (HR_max_), and maximum load achieved between maximum progressive effort tests with different amounts of muscle mass. The hypothesis of this study was that a physical exercise session performed with a larger amount of muscle mass would have greater effects on performance in executive functions than physical exercise that uses a smaller amount of muscle mass. Clarification of the effect of physical exercise performed with different amounts of muscle mass can be useful for developing physical exercise programs to prevent and treat the decline in executive functions.

## Materials and Methods

### Participants

**Figure 1:**
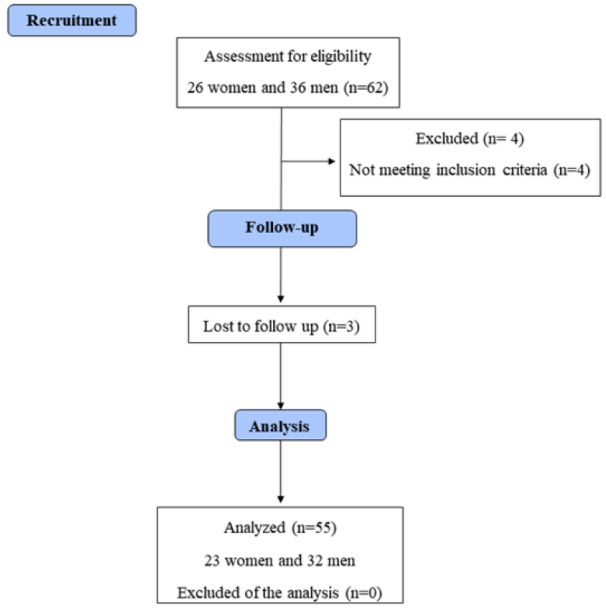
Flow diagram of the study

The sample size was calculated in G*Power version 3.1.9.7 (Franz Faul, University of Kiel, Germany) (Faul et al., 2007[20]). A sample size of 51 participants was necessary to achieve a power of 95 % and a p-value of 0.05 with a small effect size (partial eta squared: 0.05) (Takahashi and Grove, 2020[42]). Participants were recruited through social media (Instagram^®^ and WhatsApp^®^), direct contact, and announcements on our institutional website. A total of 62 healthy young adults (women and men) were recruited to participate in the study (a convenience sample). The inclusion criteria were age between 18 and 40 years and literacy. The exclusion criteria were (i) contraindications to performing physical activity (assessed using the Physical Activity Readiness Questionnaire - PAR-Q), (ii) mood and/or anxiety disorders according to a self-report made by each participant, (iii) use of stimulants (e.g., psychotropic drugs) according to a self-report made by each participant, (iv) being on menstrual period or pregnant (applicable for women), and (v) being color blind. Four participants were excluded from the study because they had an illness with repercussions on mood, and three were excluded from the study due to loss to follow-up. Therefore, the final sample comprised 55 participants (23 women and 32 men) (Table 1(Tab. 1)). The flow diagram of the study is presented in Figure 1(Fig. 1). All participants were instructed to avoid eating two hours before exercising and to refrain from caffeine, alcohol, and strenuous physical activity on the day of the experiment.

Informed consent was obtained from all participants included in this study. All experimental procedures were approved by the Federal University of Goiás Ethics Committee (CAAE approval number: 44054121.4. 0000.5083) and were performed according to the principles outlined in the Declaration of Helsinki. Furthermore, this study was registered and approved by the Brazilian Registry of Clinical Trials (ensaiosclinicos.gov.br) (registration number: RBR-9v52jkd).

### Study design

**Figure 2:**
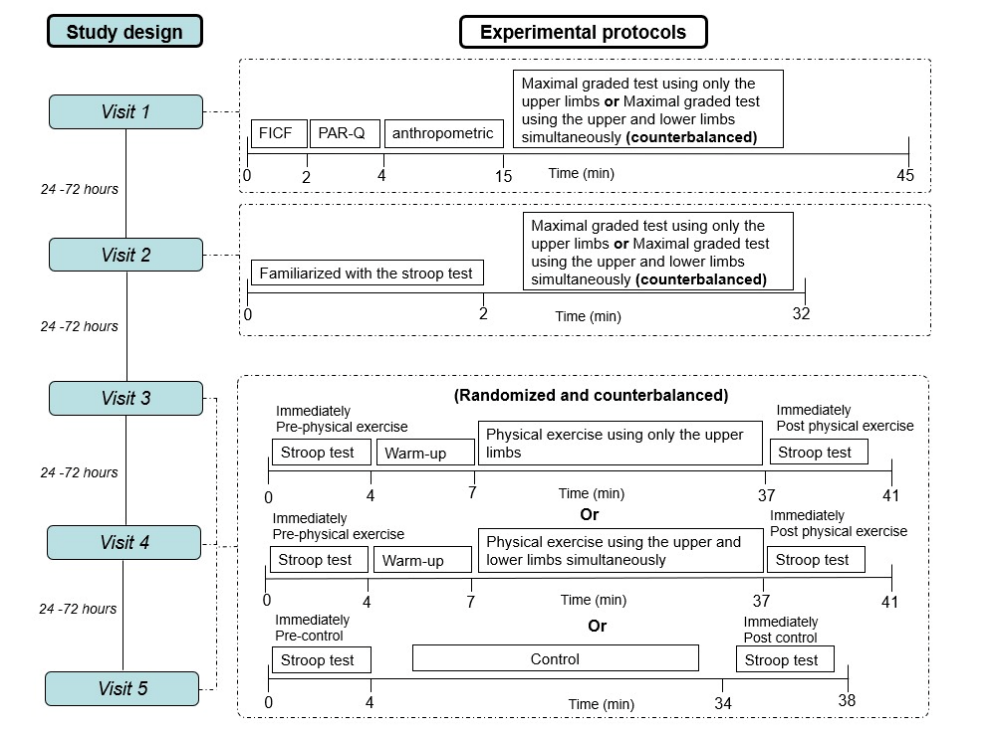
Study design. PAR-Q: Physical Activity Readiness Questionnaire. FICF: Free and informed consent form

This was an experimental study with repeated measures composed of five visits (a within-subjects design). Visits were performed with a minimum interval of 24 h between them. At the first visit, the participants underwent anamnesis, anthropometric assessment, and randomization of subsequent visits, which was performed using a website randomizer (https://www.randomizer.org/). At the first and second visits, all participants were familiarized with the cycle ergometer and performed maximal graded exercise testing with a cycling ergometer (Airbike, Movement, Brazil) using the upper and lower limbs or only the upper limbs (the order was randomly determined). On the second visit, the participants were familiarized with the Stroop test according to the guidelines (Bajaj et al., 2015[3]). This familiarization was important to minimize potential learning effects and achieve a consistent reaction time and accurate performance. At the third, fourth, and fifth visits, the participants performed a moderate-intensity continuous physical exercise session using the upper and lower limbs or only the upper limbs or a control non- physical exercise session. Each of these sessions lasted 30 min. Moderate-intensity continuous physical exercise sessions were conducted at the workload that elicited a heart rate (HR) corresponding to 64 %-76 % of HR_max_ (Garber et al., 2011[21]) obtained in maximal graded exercise testing performed with upper and lower limbs or only upper limbs. The sessions started with a three-minute warm-up to help the participants reach an HR corresponding to moderate-intensity physical exercise. All sessions were randomized and counterbalanced. During physical exercise sessions, HR and the RPE were monitored. The Stroop test was performed before and after control and physical exercise sessions. All visits were completed at the same time of day to account for circadian rhythm effects (Hill et al., 2019[24]) but separated by a minimum of 24 h. The study design is presented in Figure 2(Fig. 2).

### Experimental procedures

#### Anamnesis and anthropometric assessment

Anamnesis was performed using the Physical Activity Readiness Questionnaire (PAR-Q) (Canadian Society for Exercise Physiology, 2002[10]). This questionnaire contained seven questions to evaluate the general health condition of the participants and whether they were fit to perform physical exercise. If a participant answered “yes” to one or more questions, the participant was excluded from the study. The body mass of the participant was measured using a digital balance (Omron, HN-289, USA) to the nearest 0.1 kg, and body height was measured using a wall stadiometer (Caumaq, Brazil) to the nearest 0.1 cm. The participants' body mass indexes were calculated by dividing body mass by body height squared (kg/m²).

#### Maximal graded exercise test

All participants performed two maximal graded exercise tests, following the same protocol, on an Airbike (Movement, Brazil) on different days, separated by a minimum of 24 h and a maximum of 72 h. One test was performed using only the upper limbs and the other using both upper and lower limbs simultaneously. The protocol for the two tests was incremental (progressive and maximum) as described by the American College of Sports Medicine (2014[1]). The two maximal graded exercise tests were used to determine the HR_max_ and maximum load achieved in the tests. The order for performing the maximal graded exercise tests was counterbalanced so that 50 % of the participants first performed the test that used only the upper limbs and the other 50 % of the participants first performed the test performed with the upper and lower limbs simultaneously.

The tests started with a two-minute warm-up at 50 W, and then 25 W was added every 2 min until the participant became exhausted (Tsuk et al., 2019[45]). All participants were verbally encouraged to perform at their peak on the tests. The HR_max _achieved was used to prescribe sessions of interventions that used physical exercise. The workload of each session corresponded to the load necessary to reach an HR between 64 % and 76 % of the HR_max _(Garber et al., 2011[21]) achieved in the maximal graded exercise test.

During the tests, the participants' RPE was recorded every minute using the Borg scale, which ranges from 6 to 20, including during warm-up (Borg, 1982[7]). Likewise, HR was continuously monitored using an HR monitor (H10, Polar, Finland) placed at the xiphoid process level.

The criteria adopted for ending the tests were based on the Statement on cardiopulmonary exercise testing of the American Thoracic Society and the American College of Chest Physicians (sudden pallor, loss of coordination, mental confusion, dizziness or fainting, and signs of respiratory failure) (Ross, 2003[38]). However, the inability to maintain the requested load for more than 30 s or the participants' exhaustion would end all the tests.

#### Physical exercise intensity assessment

Physical exercise intensity was assessed by measuring participants' HR and RPE. HR was measured using an HR monitor (H10, Polar, Finland). RPE was measured using the Borg Scale (6-20) (Borg 1982[7]). The classification of physical exercise intensity followed the criteria adopted by the American College of Sports Medicine (Garber et al., 2011[21]).

#### Assessment of cognitive performance

The Stroop color-word test was used to assess cognitive performance. To this end, the EncephalApp-Stroop test for Tablets, translated and validated for Brazilian Portuguese (Bajaj et al., 2013[4], 2015[3]; Cunha-Silva et al., 2022[13]), was used to assess cognitive performance. This version was chosen because it is easy to apply and inexpensive. The internal consistency of the EncephalApp Stroop test is acceptable (intraclass coefficient = 0.832) (Bajaj et al., 2015[3]). The EncephalApp Stroop test has been widely used to evaluate the effect of physical exercise on cognitive performance (Mullen et al., 2019[32]; Hurst et al., 2020[25]; Bailey et al., 2021[2]). The Tablet used in this study was a Galaxy Tab S6 Lite (Samsung, South Korea).

The Stroop test involved two sections. The first section comprised an easier congruent “Off” state, and the second section consisted of a harder incongruent “On” state. In the Off State (congruent condition), the app randomly displayed color symbols in the form of four hashtags (####) in green, red, and blue. At the bottom of the Tablet screen, it also randomly showed the colors green, red, and blue. The participants were instructed to name the color of the symbol as quickly as possible.

In the second section, called the “On” state, an incongruent stimulus (incongruent condition) was presented to the participants (e.g., the word “red” printed in green font), for which they had to identify the color of the font rather than the word. In this section, the app randomly displayed color names in green, red, and blue. At the bottom of the Tablet screen, it also randomly showed the names of the colors green, red, and blue. The participants were instructed to identify the font color. The participants completed matches if they performed 10 stimuli marked correctly consecutively in each section. If participants made an error during any stage, the test would restart from the beginning of that stage. Furthermore, the app automatically stopped the test if more than 20 attempts were required at any stage.

The EncephalApp-Stroop test provided the following variables: time to complete five current off trials (OffTime) and time to complete five current on trials (OnTime), psychomotor speed, inhibitory control (incongruent condition), number of trials required to complete five successive trials without error (congruent and incongruent condition), and interference (difference between OffTime and OnTime). All times were presented in seconds according to the recommendations of the procedures for application of the test (Bajaj et al., 2013[4], 2015[3]), and the participants performed the 14 trials in the following order: i) two practice Off trials of “Off State”; (ii) five trials “Off State”; (iii) two practice On trials of “On State”; (iv) five trials “On State.” Two practice trials for “On State” and two practice trials for “Off State” were not used for statistical analysis. Before and after control and physical exercise sessions, participants completed the EncephalApp Stroop test to evaluate cognitive performance. Participants were instructed to respond as quickly and precisely as possible to a target presented centrally on a Tablet screen at eye level and at a distance of approximately one meter.

#### Statistical analysis

Data were analyzed using Jeffrey's Amazing Statistics Program (JASP, version 0.16.4, University of Amsterdam, Netherlands). The Kolmogorov‒Smirnov test was used to test data for normality in the *Statistical Package for the Social Sciences software* (SPSS, version 26.0, IBM Corp., USA). As the cognitive performance data did not conform to the normal distribution, the nonparametric Friedman test was used to compare performance between the control and physical exercise session times (pre vs. post). When necessary, Bonferroni post hoc analysis was used for pairwise comparisons to identify differences between treatments. Student's t test was used to compare the mean HR_max _achieved in the graded maximal exercise test between conditions (upper and lower limbs vs. lower limbs). The Wilcoxon signed rank test was used to compare the maximal workload achieved in the graded maximal exercise test. Independent Student's t-test was used to compare the height between sex. The Wilcoxon rank sum test was used to compare age, body mass and body mass index between sex. Kendall's W was used as the effect size for the Friedman test. The Kendall's W values were classified according to Cohen's as “trivial” (<0.10), “small” (0.10≤ to <0.30), “medium” (0.30≤ to <0.50), and “large” (≥0.5) (Cohen, 1988[12]). Cohen's d was used as the effect size for Student's t test (Cohen, 1988[12]). According to Cohen (1988[12]), the d values were classified as “trivial” (d < 0.2), “small” (0.20 ≤ d < 0.5), “medium” (0.5 ≤ d < 0.8), and “large” (d ≥ 0.8). The effect size used for the Wilcoxon signed rank test was the rank-biserial correlation (r_B_). The value classification was based on Pearson's correlation coefficient (r). The values were classified as “trivial” (r_B_ < 0.10), “small” (0.10 ≤ r_B_ < 0.30), “medium” (0.30 ≤ r_B_ < 0.50) and “large” (r_B_ ≥ 0.50) (Munro, 1986[33]). Normally distributed data are expressed as the mean±standard deviation, and data that deviated from the normal distribution are expressed as the median and interquartile range (IQR). The level of significance adopted for all analyses was α < 0.05.

## Results

There were no medical intercurrences during the experimental procedures of the study.

### Graded maximal exercise test

#### Heart rate, workload and rating of perceived exertion 

There was a significant difference between HR_max_ obtained using the maximal upper and lower limbs (189±10 bpm) and that obtained using only upper limbs (177±13 bpm) tests (mean difference: −12 [95 % CI: −15.06; −8.76], p < 0.001, d = −1.02 “large” [95 % CI: −1.35; −0.69]). There was a significant difference in maximal workload obtained between the test performed with upper and lower limbs (225 [IQR: 100] W) and that performed with only upper limbs (150 [IQR: 25] W) (median difference: −87.50 [95 % CI: −100; −75], p < 0.001, r_B_ = −1 “large” [95 % CI: −1; −1]). There was no significant difference in maximal RPE between the test performed with the upper and lower limbs (20 [IQR: 1]) and that performed with only the upper limbs (20 [IQR: 1]) (median difference: −0.50 [95 % CI: −1.50; 0.50], p-value = 0.36, r_B_ = −0.25 “small” [95 % CI: −0.67; 0.27]) (Table 2(Tab. 2)).

### Cognitive performance

#### Section “Off time”

**Figure 3:**
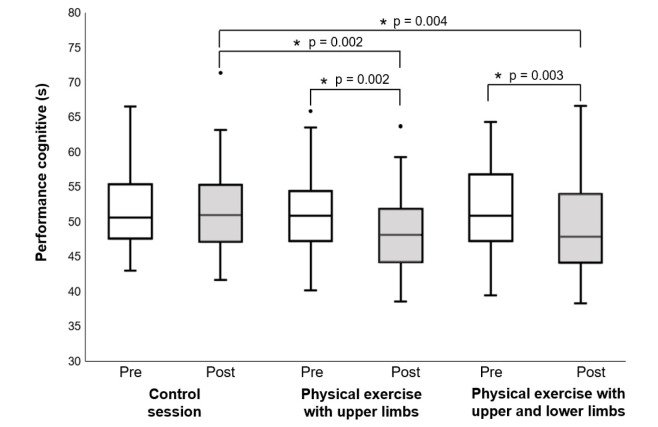
Cognitive performance before and after physical exercise sessions (physical exercise with upper limbs and physical exercise with upper and lower limbs) and control session

There was a significant time effect on cognitive performance (Friedman test, χ^2^ [5] = 46.211, p-value < 0.001, Kendall's W = 0.168 “small”) (Figure 3(Fig. 3)). Cognitive performance after physical exercise with the upper limb was higher than that measured before the upper limb physical exercise (Bonferroni's post hoc analysis, p-value = 0.002). Similarly, cognitive performance after physical exercise with the upper and lower limbs was higher than that measured before physical exercise with the upper and lower limbs (Bonferroni's post hoc analysis, p-value = 0.003). However, there was a significant difference in cognitive performance before and after the control session (Bonferroni's post hoc analysis, p-value = 1.000). Furthermore, cognitive performance after both physical exercise conditions was higher than that measured after the control session (Bonferroni's post hoc analysis, p-value = 0.002 and 0.004 for physical exercise with upper limbs and physical exercise with upper and lower limbs, respectively). In contrast, there was no significant difference in cognitive performance between the two physical exercise conditions (Bonferroni's post hoc analysis, p-value = 1.000).

#### Section “On time”

**Figure 4:**
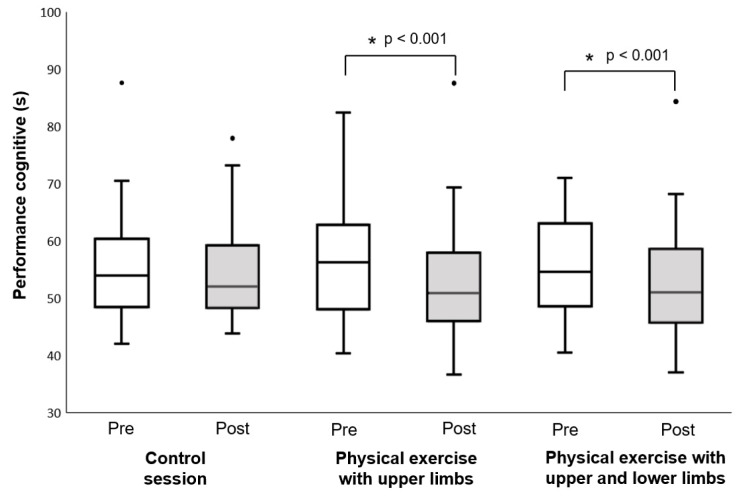
Cognitive performance before and after physical exercise sessions (physical exercise with upper limbs and physical exercise with upper and lower limbs) and control session. There was a significant difference between times (p-value < 0.005).

The Friedman test detected a significant time effect on cognitive performance (χ^2^ [5] = 43.332, p-value < 0.001, Kendall's W = 0.158 “small”) (Figure 4(Fig. 4)). Cognitive performance after physical exercise with upper limbs was higher than that measured before the physical exercise (Bonferroni's post hoc analysis, p-value < 0.001). Additionally, cognitive performance after physical exercise with both upper and lower limbs was higher than that measured before the physical exercise (Bonferroni's post hoc analysis, p-value < 0.001). However, there was no difference in cognitive performance before and after the control session (Bonferroni's post hoc analysis, p = 1.000). Furthermore, cognitive performance after physical exercise with upper limbs only did not differ from that measured after the control session (p-value = 0.175). Similarly, cognitive performance after physical exercise with the upper and lower limbs was not significantly different from that measured after the control session (Bonferroni's post hoc analysis, p-value = 0.130). Additionally, cognitive performance after physical exercise with the upper and lower limbs did not differ from that measured after physical exercise with upper limbs (Bonferroni's post hoc analysis, p-value = 1.000).

#### Number of matches in the “Off State” section 

The Friedman test detected no significant time effect on the number of matches (χ^2^ [5] = 7.925, p-value = 0.160, Kendall's W = 0.029 “trivial”). Table 3(Tab. 3) shows values of the number of matches of the congruent condition.

#### Number of matches in the section “On state” (incongruent condition)

The Friedman test did not detect a significant time effect on the number of matches (χ^2^ [5] = 6.664, p-value = 0.247, Kendall's W = 0.024 “trivial”). Table 4(Tab. 4) shows the values of the number of matches of the congruent condition.

#### Condition congruent less incongruent (interference effect)

The Friedman test detected a significant time effect on the interference effect (χ^2^ [5] = 12.672, p-value = 0.027, Kendall's W = 0.046 “trivial”). However, Bonferroni's post hoc analysis did not show statistical significance for any of the peer comparisons (p-value > 0.005). Table 5(Tab. 5) shows the values of the interference effect. (Raw data are presented in the Supplementary data file).

## Discussion

The main aim of this study was to characterize and compare the acute effect of muscle mass involved in physical exercise of moderate intensity on the performance of executive functions. Second, we compared the RPE, HR_max_, and maximum load achieved between maximum progressive effort tests with different muscle mass. We found that both physical exercise interventions improved Stroop task performance in a congruent trial. Additionally, physical exercise intervention similarly improved Stroop task performance in an incongruent trial but without any difference from the resting control condition. Therefore, the results partially confirmed our initial hypothesis.

### Maximal graded exercise test

The results obtained from a maximal graded exercise test are particularly useful for assessing the functional capacity of the subject, monitoring the effects of a training program, detraining, and the presence of diseases (Takken et al., 2019[43]). Although we did not evaluate the oxygen consumption responses, we evaluated the HR and used the HR_max _to prescribe moderate physical exercise intensity. Additionally, the comparison of the variables between the tests was important for demonstrating that the protocol used evoked different physiological responses as a function of the amount of muscle mass involved in the task. As expected, we found significant differences in HR_max_ and maximum workload between the tests; these variables were significantly higher for the test involving the upper and lower limbs simultaneously.

Regarding HR_max_, our results are in agreement with those of Nagle et al., (1984[34]), who found that the combination of 10 % upper limbs/90 % lower limbs evoked a higher HR_max_ than the test using 100 % upper limbs. However, the HR_max_ obtained for the combination of 10 % upper limbs/90 % lower limbs was not significantly different from that obtained for the test performed with 100 % lower limbs and the test performed with lower limbs. These results suggest that the higher the contribution of the upper limbs (from 20 % upper limbs/80 % lower limbs), the lower the HR_max_.

Regarding the maximum workload achieved in the maximal graded exercise tests, Nagle et al., (1984[34]) reported that the highest loads were achieved for the combinations of 10 % upper limbs/90 % lower limbs and 20 % upper limbs/80 % lower limbs, without a significant difference between the two combinations. However, these two combinations evoked a maximum workload significantly higher than that of all the other test combinations (100 % upper limbs [125 ± 6 W], 100 % lower limbs [239 ± 11 W], and 30 % upper limb/70 % lower limb [229 ± 15 W]). Likewise, the highest maximal oxygen uptake was obtained for the 10 % upper limb/90 % lower limb combination. 

### Executive performance

We found that a single 30-min session of moderate-intensity aerobic physical exercise, regardless of the amount of muscle mass involved in its performance, significantly decreased the reaction time to complete the Stroop task (congruent trial) immediately after physical exercise interventions (without a significant difference between conditions) relative to the reaction time before physical exercise interventions. In contrast, the resting control session did not alter executive performance. Furthermore, the reaction time after both physical exercise interventions was lower in the congruent trial than in the postresting control session. Notably, the improvement (decrease) in the times to complete the congruent trial after the two interventions with physical exercise occurred without compromising the accuracy of performing this task because the number of attempts needed to complete the section showed no statistically significant difference (before vs. after).

Regarding the incongruent trial, reaction times after interventions (physical exercise and control) were not significantly different. However, there was a statistically significant decrease in the reaction time to complete the section in the two interventions with physical exercise relative to the pre- and post-times, without compromising the precision, a fact that did not occur in the resting control intervention. This implies that, when comparing the pre- and post-times, there was only improvement in interventions with aerobic physical exercises performed with different amounts of muscle mass involved in their performance. However, when we compared the post-reaction time of all interventions, they did not differ.

Several previous studies have reported that moderate-intensity aerobic physical exercise can improve performance on several tests that assess executive functions (including different versions of the Stroop test) (Ludyga et al., 2016[27]). For example, Rattray and Smee (2013[37]) found that physical exercise caused a statistically significant decrease in reaction time without a decrease in accuracy for performing the task relative to the control condition without physical exercise. Furthermore, when comparing the reaction times in the 10^th^ minute of each intervention, the intervention with physical exercise showed a reduction of approximately 3 % in reaction time compared to the control.

Our results are somewhat in line with those of Tam (2013[44]), who reported that the physical exercise of climbing stairs improved (decreased) the time to perform the Stroop test immediately after the physical exercise intervention. Additionally, there was also a reduction in errors (improvement in accuracy) when performing the test. Decreases in errors (precision) were greater in incongruent trials than in congruent trials. There was no significant improvement in the test performed after the resting control situation. The author concluded that a single stair-climbing physical exercise session promoted improvements in the processing of executive functions (mainly for tests, our results are in line with those of Hill et al., (2019[24]), who reported that cycling physical exercise performed only with upper limbs and cycling exercise performed with lower limbs caused similar benefits for executive functions because there was a significant and moderate reduction in reaction time in congruent and incongruent trials without impairing accuracy.

Although it is not clear how aerobic physical exercise affects executive functions, some previous studies suggest that physical exercise can cause neurophysiological changes that influence brain functioning (Basso and Suzuki, 2017[5]; Mehren et al., 2019[30]). For example, physical exercise can increase cerebral blood flow in the prefrontal region of the brain, which is highly important for executive functions. Additionally, physical exercise can also increase the release of neurotransmitters such as catecholamines, dopamine, and norepinephrine and can also promote increases in BDNF (Chang et al., 2012[11]; Basso and Suzuki, 2017[5]), which influence the functioning of the brain. Although such neurophysiological changes were not evaluated in this study, it is reasonable to assume that they may be responsible for the positive changes found here. Furthermore, it is also legitimate to assume that the amount of muscle mass involved in a physical exercise does not impact neurophysiological responses differently, contrary to what occurs with the cardiovascular, respiratory, and muscular systems.

Another possible explanation of how physical exercise improves the performance of executive functions, from a psychological point of view, is that physical exercise causes excitation (at appropriate levels) that improves resource allocation to mental processes, thereby facilitating the cognitive process (Yanagisawa et al., 2010[50]; Byun et al., 2014[9]; Tsukamoto et al., 2016[46]; Mandolesi et al., 2018[29]). Likewise, although we did not measure this arousal in this study, it is reasonable to assume that the two types of physical exercise invoke the same degree of arousal, which could explain why the physical exercise interventions improved performance on the Stroop test.

Moreover, no difference was observed between the two types of physical exercise relative to the control on “congruent minus incongruent.” Takahashi and Grove (Takahashi and Grove, 2020[42]) stated that interference may have low reliability for research with a design similar to that used in this study, since this variable may present a lower interclass correlation index than congruent and incongruent trials. Therefore, the use of this variable can mask the significance of the effect of a single physical exercise session on the performance of the Stroop test. We suggest that analyzing and comparing the results of congruent and incongruent trials in the Stroop task (as done in the present research) may be a better approach for a more comprehensive understanding of the effect of physical exercise on executive functions.

### Future perspectives

Further studies can evaluate the effects of other types of physical exercise (e.g., high-intensity interval physical exercise and resistance physical exercise) performed with different amounts of muscle mass on cognitive function. Furthermore, caution is needed in extrapolating the findings of this study to other populations, such as athletes, elderly people, and people with clinical cognitive disorders. Therefore, further studies investigating these populations are needed. Finally, it is desirable to conduct studies investigating the chronic effects of physical exercises performed with different amounts of muscle mass on executive functions.

### Study limitations

This study had some limitations. The absence of biochemical and physiological measures, such as serotonin, endorphin, BDNF and cerebral blood flow, compromised the reliability of the results. However, we believe that these limitations do not warrant dismissal of the conclusions presented here.

## Conclusions

The findings of this study suggest that for purposes of improving executive function, moderate-intensity continuous aerobic physical exercise performed using the upper limbs or using the upper and lower limbs simultaneously improves executive functions. Therefore, we recommend that health professionals consider moderate-intensity aerobic continuous physical exercise with less or more muscle mass to improve cognitive function.

## Declaration

### Acknowledgments

We would like to thank all the participants who volunteered to participate in this study. This work was supported by the Fundação de Amparo à Pesquisa do Estado de Goiás-FAPEG/Brazil (Grant number 201210267001056), Conselho Nacional de Desenvolvimento Científico e Tecnológico-CNPq (Grant number 405096/2016-0), and Coordenação de Aperfeiçoamento de Pessoal de Ensino Superior-CAPES/Brazil (Grant number Finance code 001). CABL and MSA are productivity fellowships at the Conselho Nacional de Desenvolvimento Científico e Tecnológico (CNPq, Brazil). RLV is a productivity fellowship at the Espírito Santo Research and Innovation Support Foundation (FAPES) agency (Public Notice N° 06/2021-Bolsa Researcher Capixaba).

### Authors' contributions

MJM and CABL: study concept and design. MJM: data acquisition; MJM and VNO: data analysis, interpretation, and article preparation. RBV, RMA, MHC, GCTC, MSA, CAV, RLV, BK, KW, and CABL: critical revision of the article. All the authors have read and approved the final article.

### Conflict of interest

No competing financial interests exist.

## Supplementary Material

Supplementary data

## Figures and Tables

**Table 1:**
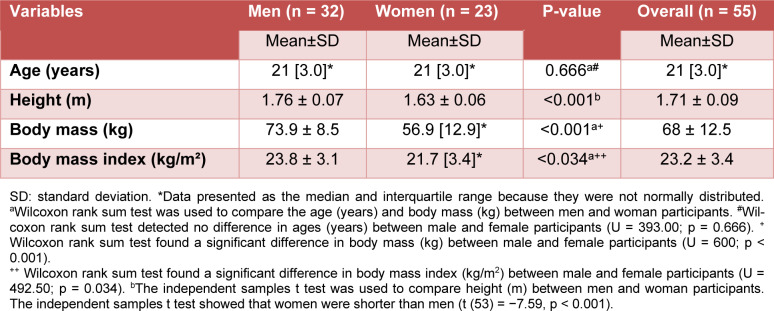
Characteristics of the participants

**Table 2:**
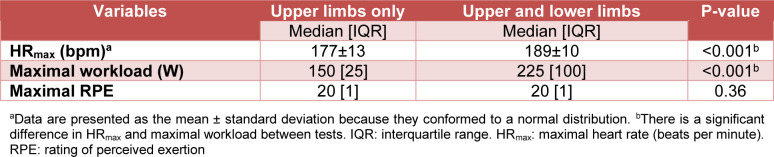
Values of the HR_max_, maximal workload, and maximal RPE achieved in the graded maximal exercise test performed with upper limbs and with upper and lower limbs (n = 55)

**Table 3:**
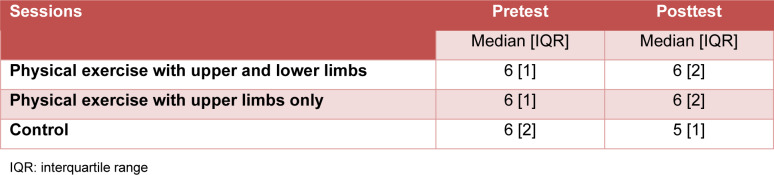
Number of matches of the section “Off State” (congruent condition) (n = 55)

**Table 4:**
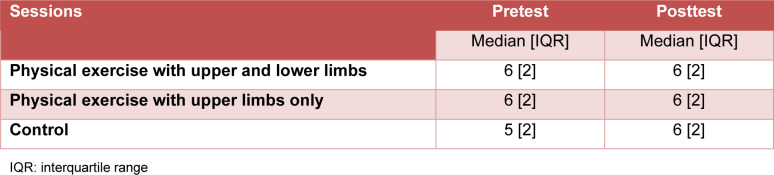
Number of matches of the section “On state” (incongruent condition) (n = 55)

**Table 5:**
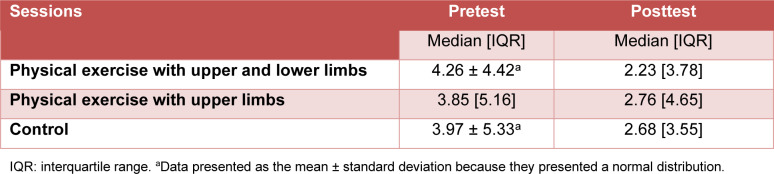
Condition congruent less condition incongruent in seconds (n = 55)
